# uPAR-expressing melanoma exosomes promote angiogenesis by VE-Cadherin, EGFR and uPAR overexpression and rise of ERK1,2 signaling in endothelial cells

**DOI:** 10.1007/s00018-020-03707-4

**Published:** 2020-11-25

**Authors:** Alessio Biagioni, Anna Laurenzana, Beatrice Menicacci, Silvia Peppicelli, Elena Andreucci, Francesca Bianchini, Daniele Guasti, Paolo Paoli, Simona Serratì, Alessandra Mocali, Lido Calorini, Mario Del Rosso, Gabriella Fibbi, Anastasia Chillà, Francesca Margheri

**Affiliations:** 1 Department of Experimental and Clinical Biomedical Sciences, Section of Experimental Pathology and Oncology, University of Florence, Viale G.B. Morgagni, 50, 50134 Florence, Italy; 2 Department of Experimental and Clinical Medicine, University of Florence, Viale Pieraccini 6, 50139 Florence, Italy; 3 Nanotecnology Laboratory, National Cancer Research Centre, IRCCS “Giovanni Paolo II”, Viale Orazio Flacco, 65, 70124 Bari, Italy

**Keywords:** uPA/uPAR system, Exosomes, Melanoma cells, Angiogenesis, Endothelial cells

## Abstract

Exosomes (Exos) have been reported to promote pre-metastatic niche formation, proliferation, angiogenesis and metastasis. We have investigated the role of uPAR in melanoma cell lines-derived Exos and their pro-angiogenic effects on human microvascular endothelial cells (HMVECs) and endothelial colony-forming cells (ECFCs). Melanoma Exos were isolated from conditioned media of A375 and M6 cells by differential centrifugation and filtration. Tunable Resistive Pulse Sensing (TRPS) and Nanoparticle tracking analysis were performed to analyze dimension and concentration of Exos. The CRISPR–Cas 9 technology was exploited to obtain a robust uPAR knockout. uPAR is expressed in melanoma Exos that are internalized by HMVECs and ECFCs, enhancing VE-Cadherin, EGFR and uPAR expression in endothelial cells that undergo a complete angiogenic program, including proliferation, migration and tube formation. uPAR loss reduced the pro-angiogenic effects of melanoma Exos in vitro and in vivo by inhibition of VE-Cadherin, EGFR and uPAR expression and of ERK1,2 signaling in endothelial cells. A similar effect was obtained with a peptide that inhibits uPAR–EGFR interaction and with the EGFR inhibitor Gefitinib, which also inhibited melanoma Exos-dependent EGFR phosphorylation. This study suggests that uPAR is required for the pro-angiogenic activity of melanoma Exos. We propose the identification of uPAR-expressing Exos as a potentially useful biomarker for assessing pro-angiogenic propensity and eventually monitoring the response to treatment in metastatic melanoma patients.

**Electronic supplementary material:**

The online version of this article (10.1007/s00018-020-03707-4) contains supplementary material, which is available to authorized users.

## Introduction

Melanoma is the deadliest skin cancer, with stage IV melanoma patients having a 5-year survival rate of less than 15% [1]. Despite significant advances in melanoma therapy, invasive melanoma incidence continues to rise worldwide faster than any other cancer [2] and it is responsible for approximately 70% of skin cancer-associated mortality [3]. The major cause of melanoma mortality is metastasis to distant organs, in lungs and brain [4]. In spite of many advances in understanding the cellular and molecular interactions within the tumor microenvironment, the underlying mechanism contributing to the generation of the pre-metastatic niche at distal sites remains elusive. Emerging evidence suggests that cancer-derived extracellular vesicles (EVs) play a major role not only in conditioning the tumor microenvironment but also in preparing the “soil” of the pre-metastatic niche for metastasis. [5, 6]. There are several types of EVs: microvesicles (MVs), apoptotic bodies and exosomes (Exos). Exos are nano-sized (40–150 nm) membrane-bound vesicles that originate from the late endosomal trafficking, are gathered intracellularly into multivesicular bodies (MVBs) and released by fusion with the plasma membrane [7]. They are critical mediators of intercellular communication between tumor and stromal cells via their biologically active payload, including proteins, lipids and metabolites, RNA and DNA [8, 9]. Exos are reported to promote proliferation, invasion, and chemoresistance of cancer cells, to stimulate reprogramming of stromal cells to Cancer-Associated Fibroblasts (CAFs) and to promote angiogenesis which is critical for tumor cells release in the circulation and their spread to distant sites [10–13].

The uPA/uPAR system components (urokinase-type plasminogen activator, uPA; uPA receptor, uPAR; uPA inhibitor type-1, PAI-1) are actually considered important prognostic and predictive markers of malignancy [14]. Several malignant tumors show a positive correlation between uPAR levels, a more aggressive phenotype and a poor prognosis [15]. We have previously shown that uPAR is strongly up-regulated in A375 and in metastasis-prone M6 melanoma cells with respect to normal melanocytes [16]. uPAR overexpression in melanoma cells controls an invasive and glycolytic phenotype depending on alpha 5-beta1 integrin -mediated uPAR connection with EGFR [17]. We have also demonstrated that uPAR acquires a primary importance in vemurafenib resistance in tumors harboring the BRAF V600E mutation: high levels of uPAR and EGFR are associated with a lower sensitivity to vemurafenib [18]. At the same time, also the soluble urokinase-type plasminogen activator receptor (suPAR) has been proposed as a biomarker of tumor progression in several malignant tumors such as non-small-cell lung cancer [19], advanced breast cancer [20], colorectal cancer [21], ovarian cancer [22] and prostate cancer [23] and have been correlated with a poor prognosis.

Based on the above, in the current study, we aimed at addressing the role of uPAR in melanoma-derived Exos. We started to investigate the expression of uPAR both in ectosomes and Exos and the pro-angiogenic effects of melanoma Exos on human endothelial colony-forming cells (ECFCs) and on human microvascular endothelial cells (HMVECs). We have observed a quantitative increase of VE-Cadherin, EGFR and uPAR, along with a rise of EGFR phosphorylation and ERK1,2 signaling. We have further assessed the effects of CRISPR–Cas 9-mediated uPAR knockdown and of its rescue on the pro-angiogenic activity of melanoma-derived Exos in vitro and in vivo.

## Materials and methods

### Cell lines and culture conditions

The human melanoma cell line A375 (MITF wild type, BRAF V600E, NRAS wild type) was obtained from American Type Culture Collection (Manassas, VA) and was grown in Dulbecco’s modified Eagle’smedium (DMEM, Euroclone, Milano, Italy) containing 2 mM glutamine, 100 UI/ml penicillin, 100 μg/ml streptomycin and 10% FBS (Euroclone, Milano, Italy). A375–M6 melanoma cells (M6) were isolated from lung metastasis of SCID bg/bg mice i.v. injected with A375 cells and grown in the same conditions of A375. A375 and M6 were independently validated by STR profiling by the DNA diagnostic centre BMR Genomics (Padova, Italy). Cells were amplified, stocked, thawed and were kept in culture for a maximum of 4 months. ECFCs were isolated from > 50 ml human umbilical cord blood (UCB) of healthy newborns as described previously [24], were selected as CD45^−^, CD34^+^, CD31^+^, CD105^+^, ULEX^+^, vWF^+^, KDR^+^ cells [24] and were grown in EGM-2 culture medium (Lonza), supplemented with 10% FBS. Human Microvascular Endothelial Cells (HMVECs) were purchased from Lonza and were grown in the same conditions of ECFCs.

### Exosomes isolation

Exos isolation protocol was modified from the previously published methods [25, 26]. See details in Supplemental Methods.

### TRPS (Tunable Resistive Pulse Sensing) analysis

The size distribution and concentration of Exos were measured by TRPS analysis using a qNano platform with an NP100-rated nanopore (Izon Science, UK). Exosomal samples were diluted 1000-fold with PBS and measured three times. Data processing and analysis were carried out on the Izon Control Suite software (Izon Science, UK).

### Nanoparticle tracking analysis

Nanoparticle tracking analysis was performed on a NanoSight NS300 (Malvern Panalytical, Westborough, MA, USA) equipped with a 488 nm excitation laser and an automated syringe sampler. NanoSight technology calculates size based on the relationship between Brownian motion and hydrodynamic diameter through the Stokes–Einstein equation. Exosomal samples were diluted 1:500 in PBS and loaded into 1 ml syringes. CSV files generated by NTA by software v3.2 were used for computational analysis.

### Transmission electron microscopy (TEM)

Negative staining technique was employed to visualize the Exos. An enriched exosomal suspension in filtered DPBS (Dulbecco's phosphate-buffered saline) was dispensed on carbon-coated electron microscopy grids on parafilm and left to absorb for 10 min at room temperature then transferred to a drop of Uranyless® solution for 1 min and left to air dry. Excess stain was blotted away. Imaging was performed using a JEOL 100CX II transmission electron microscope (TEM) at 100 kV.

### PKH67 labeling of Exos and exosomal uptake into recipient cells

Melanoma-derived Exos were collected from 80 ml of culture medium as described above. Exos were labeled using PKH67 Fluorescent Cell Linker kit (Sigma-Aldrich, St. Louis, MO) according to the manufacturer's instructions. Exos uptake was measured on a FACSCAN LSRII (Becton–Dickinson, excitation = 490 nm, emission = 502 nm) as previously described [27]. See details in Supplemental Methods.

### Immunofluorescence confocal microscopy

Immunofluorescence was performed as previously described [24, 28]. See details in Supplemental Methods.

### Cell proliferation assay

12 × 10^4^ HMVECs and ECFCs/well were seeded in six-well plates. The day after plating, the standard culture medium was substituted with EBM plus 2% FBS in the presence or absence of A375- and M6-derived Exos (20 µg/ml) (indicated as A375-Exos and M6-Exos, respectively) at final concentration of 20 µg/ml. Cell proliferation was evaluated by cell counting at 24 h, 48 h, 72 h and 96 h.

### Wound healing assay

12 × 10^4^ HMVECs and ECFCs/well were seeded in six-well plates and grown to confluency. The standard culture medium was then substituted with EBM plus 2% FBS in the presence or absence of A375-Exos and M6-Exos at final concentration of 20 µg/ml and a wound was produced in each well with a 20-μl micropipette tip. Microphotographs of the wound were taken at time 0, 6 h and 24 h. Images were analyzed with the Image J MRI Wound healing software and reported as the percentage of the healing compared to initial wound area.

### Invasion assays in Boyden chambers

Spontaneous invasion experiments were performed in Boyden chambers as previously described [24], with wells separated by 8-µm-pore size polycarbonate filters coated with Matrigel (50 µg/filter). See details in Supplemental Methods.

### Capillary morphogenesis

In vitro capillary morphogenesis was performed as described [24], in tissue culture wells coated with Matrigel (BD Biosciences). ECFCs and HMVECs were resuspended (18 × 10^3^ /well in 96-well plates) in EBM 2% FBS in the presence or absence of A375-Exos and M6-Exos (20 µg/ml) and incubated for 6 h at 37 °C, 5% CO_2_. Results were quantified at the end of experiment with Angiogenesis Analyzer tool of Image J software, measuring the number of junctions, branches, tubules, total length and total tubule length. Six to nine photographic fields from three plates were analyzed for each point.

### Western Blot analyses

Ectosome, Exos and cell aliquots of A375 and M6, as well as cell aliquots of control and Exos-treated ECFC and HMVEC cultures were processed for Western Blotting analyses. See details in Supplemental Methods.

### Cell treatment with M25 integrin antagonist peptide and Gefitinib

Inhibition of uPAR–integrin interaction was obtained with the M25 peptide as previously described [17, 18], while EGFR phosphorylation was inhibited with Gefitinib, a specific inhibitor of the EGFR tyrosine kinase. See details in Supplemental Methods.

### siRNA for uPAR gene knockdown

Targeting and not-targeting siRNAs were obtained from Dharmacon (Carlo Erba Reagents, Milan, Italy). Specific silencing of uPAR gene was performed, as previously described [17, 18]. See details in Supplemental Methods.

### Double Nickase Cas9 *PLAUR* gene knockout

A complete *PLAUR* gene knockout was obtained, as previously described [29], by transfection of A375 and A375-M6 with two CRISPR/Cas9 D10A plasmids, each one bearing a specific sgRNA designed by the manufacturer to generate a double strand break in uPAR exon 3. For uPAR expression rescue experiments, cells were stably transfected using an Okayama–Berg vector containing uPAR cDNA and selected with G418 as resistance marker (0.5 mg/ml) as previously reported [29, 30].

### In vivo Matrigel plug assay

All procedures involving animals were performed in accordance with the ethical standards and according to the Declaration of Helsinki and to national guidelines approved by the ethical committee of Animal Welfare Office of Italian Health Ministry and conformed to the legal mandates and Italian guidelines for the care and maintenance of laboratory animals. Five hundred µl of Matrigel (BD Biosciences) mixed with (20 µg/ml) Exos from wild type, uPAR ko- and uPAR rescued M6 was injected subcutaneously in the ventral region of nude mice (12 mice, 4 animals for each condition) (Charles River). After 7 days, the Matrigel was excised and then fixed with formalin overnight, embedded in paraffin, and sectioned to obtain slides. The plugs were stained with hematoxylin and eosin (H&E) and visualized using the Zeiss inverted microscope (Zeiss, Germany). In vivo neovascularization was quantified by blood vessel density using Image J software.

### Statistical analysis

Statistical analyses of the data were performed using one-way ANOVA, and *p* ≤ 0.05 was considered a statistically significant difference; while *p* ≤ 0.01, a very statistically significant difference.

## Results

### Isolation and characterization of melanoma-derived Exos

**Fig. 1:**
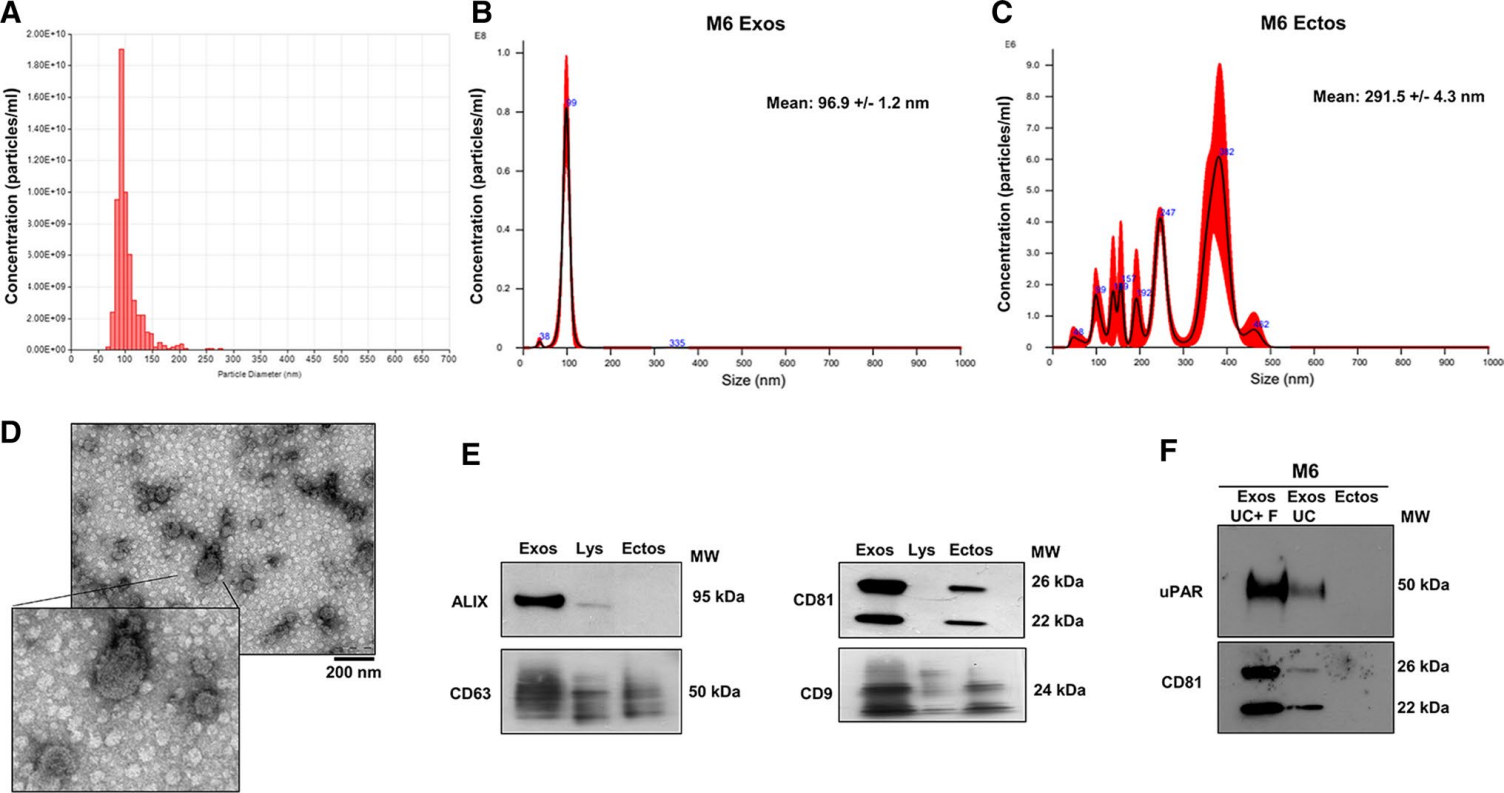
Characterization of M6-derived exosomes and ectosomes. **a** Particles size distribution and concentration by TRPS of M6-Exos. **b**, **c** Nanoparticles Tracking Analysis of exosomes (Exos) and ectosomes (Ectos), respectively, by Nanosight technology. **d** Morphology and dimension of M6-Exos under a transmission electron microscopy. Scale bar: 200 nm. **e** Western blotting analysis of exosomal marker proteins (including ALIX, CD63, CD81, CD9). **f** Western Blotting of uPAR in M6-Exos and ectosomes. CD81 was used as loading control. *MW* molecular weight; *Exos* exosomes; *Lys* total lysate; *Ectos* Ectosomes; *UC* ultracentrifugation; *F* filtration

Melanoma-derived Exos were isolated from culture media (CM) of A375 and M6, the metastatic clone of A375, after 48 h incubation in serum-free media by differential centrifugation and filtration. In order to identify the purified Exos, we characterized our population by TRPS and Nanoparticle tracking analysis. TRPS analysis (Fig. 1a) showed the distribution and concentration of M6-Exos. The size of M6-Exos was approximately ranging from 50 to 150 nm, concordant with the previously reported exosomal size distribution [26]. Similar results were obtained with Nanoparticle tracking analysis performed by Nanosight (mean: 96.9 ± 1.2 nm**)** (Fig. 1b). Nanoparticle tracking analysis of ectosomes (Fig. 1c) showed several components with different dimension. The mean size of this distribution was 291.5 ± 4.3 nm according to the previous observations [31]. TEM analysis revealed, as evident in Fig. 1d, that M6-Exos have a round-, cup-shaped morphology with a diameter ranging from 50 to 150 nm, consistent with the data of TRPS and Nanoparticle tracking. Western Blotting analyses indicate that M6-Exos were positive for the characteristic exosomal surface marker proteins (CD9, CD63, and CD81, members of tetraspanin family) and ALIX, a component of Multivesicular Body (MVB), expressed only in Exos [10], confirming the efficacy of purification methods (Fig. 1e). Similar results were obtained in A375-Exos (Supplementary Fig. S1, panels A, B, C and D).

### uPAR expression in melanoma-derived Exos

uPAR is an important prognostic and predictive marker in melanoma progression [16, 17]. We have previously shown that uPAR is strongly up-regulated in A375 and M6 melanoma cells with respect to human melanocytes [16]. To investigate the expression of uPAR in melanoma-derived EVs, we analyzed uPAR levels in both ectosomes (collected from CM after 12000×*g* centrifugation) and Exos (collected from CM after filtration and 100,000×*g* ultracentrifugation). Western Blotting analyses (Fig. 1f) show that uPAR is present only in Exos but not in ectosomes and, in particular, we observed an enrichment of uPAR in the smaller vesicles obtained by ultracentrifugation combined with filtration compared to those collected after ultracentrifugation only. CD81 was used as loading control. Similar results were obtained in A375 (Supplementary Fig. S1, panel E).

### Internalization of A375- and M6-Exos by HMVECs and ECFCs

**Fig. 2:**
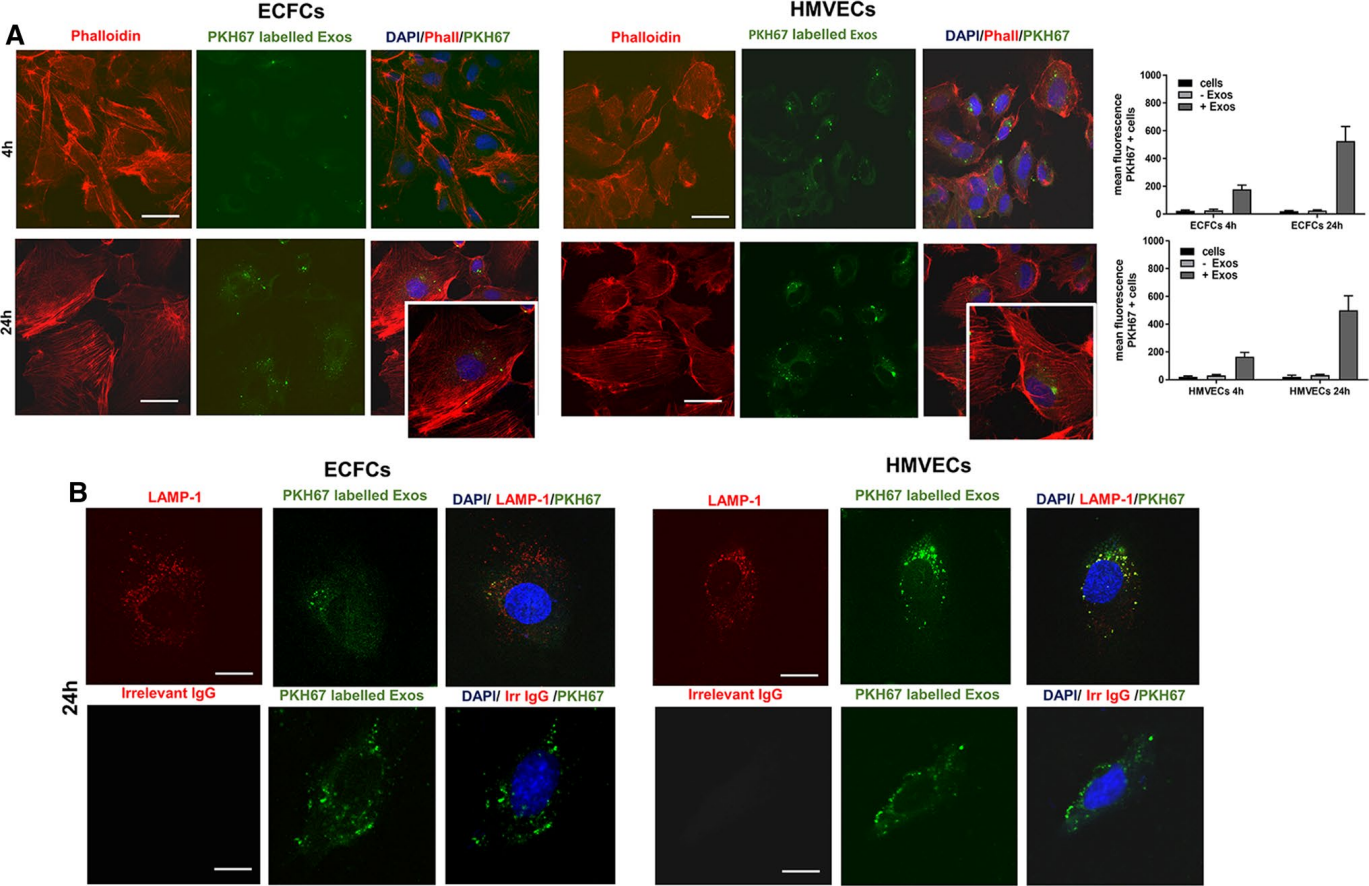
Internalization of M6-Exos into ECFCs and HMVECs. **a** Localization by confocal microscopy analysis of PHK67-labeled M6-Exos in ECFCs and HMVECs stained with TRITC-labeled phalloidin. The green-labeled Exos were visible in the perinuclear region of HMVECs and ECFCs. Scale bar: 20 µm. Histograms on the right represent the PHK67 mean fluorescence intensity of ECFCs and HMVECs after 4-h and 24-h incubation with stained exosomes (+(Exos) or an exosome-negative control (-Exos). **b** Confocal microscopy analysis of PHK67-labeled M6-Exos and LAMP-1, a lysosome marker. Scale bar: 10 µm

After melanoma Exos characterization, we assessed the effects of A375- and M6-Exos on the angiogenic activities of endothelial cells in vitro. First, we determined whether melanoma Exos could be internalized into endothelial cells. To this purpose, A375- and M6-Exos were labeled by a green fluorescent lipophilic dye (PHK67) and HMVECs and ECFCs were incubated with green Exos for 4 and 24 h, then stained with phalloidin and analyzed by immunofluorescence for LAMP-1, a lysosome marker. After 24 h, the labeled M6-Exos were evident in the perinuclear region of endothelial cells at the lysosomal compartment, indicating that M6-Exos were efficiently internalized into ECFCs and HMVECs. (Fig. 2a and b. To quantify the exosome uptake in endothelial cells, we performed flow cytometry analysis. The results (Fig. 2a, on the right) confirmed our previous microscopy observations and showed that the uptake of exosomes was time dependent. Indeed, the mean fluorescence intensity of PHK67 + endothelial cells results increased after 24-h incubation with M6-labeled exosomes. Similar results were obtained with A375-Exos (Supplementary Fig. S2).

### Pro-angiogenic effects of A375- and M6-Exos on endothelial cells

**Fig. 3:**
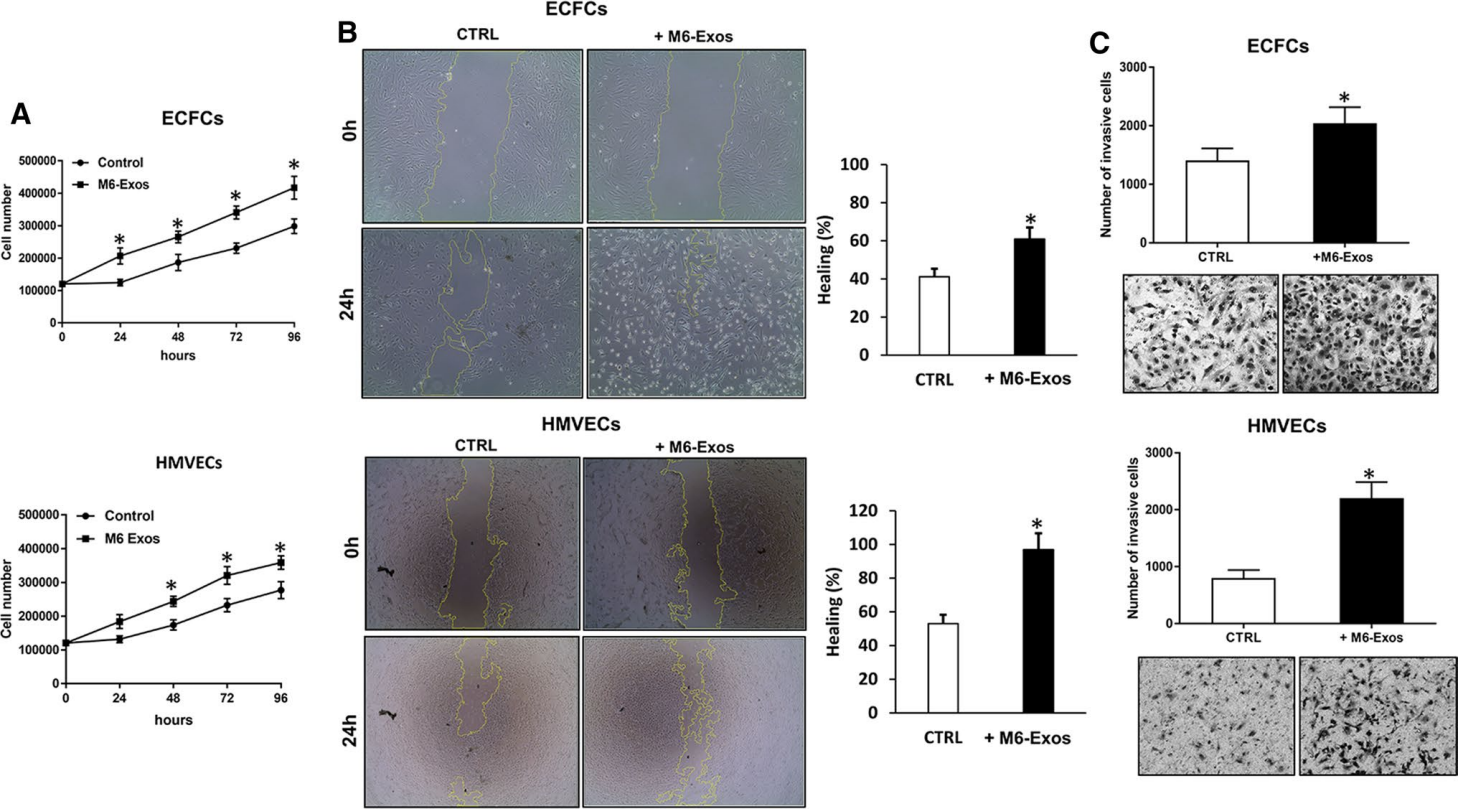
Effects of M6-Exos on proliferation, migration and invasion of ECFCs and HMVECs. **a** Cell proliferation in the presence or absence of M6-Exos analyzed by cell counting at 24, 48, 72 and 96 h. Results are reported as mean ± SD of three different experiments. **b** Wound healing assays in the presence or absence of M6-Exos. Microphotographs (× 10) at 0 and 24 h from the wound are shown. Histograms represent the percentage of the healing (the mean of three different experiments ± *SD* is reported) measured after incubation with or without M6-Exos. * shows statistical significance (*p* < 0.05) compared to untreated cells. **c** Spontaneous invasion. 2.5 × 10^4^ cells were suspended in the reference media in the presence or absence of M6-Exos and placed in the upper well. Fresh EBM was placed in the lower well. Data are reported as the number of migrated cells after incubation with M6-Exos suspended in EBM, compared to those migrated after incubation with fresh EBM alone. All histograms represent the mean of three different experiments ± SD. Representative microphotographs (× 10) of migrated cells are shown under the respective histogram

To explore the functional role of melanoma Exos on angiogenesis, HMVECs and ECFCs were treated with A375- and M6-Exos for a series of in vitro angiogenesis-related assays: proliferation, migration and invasion. The effect of M6-Exos on the proliferation was analyzed by cell counting at 24, 48, 72 and 96 h. M6-Exos significantly enhanced (about 50% for ECFCs and 40% for HMVECs, respectively) the proliferation of HMVECs and ECFCs compared to untreated cells at any time of observation (Fig. 3a). A scratch wound healing assay was performed to assess the effect of M6-Exos on the migration of endothelial cells. The results of wound closure quantification (% of healing) showed in Fig. 3b demonstrated that exosomal treatment remarkably increased the motility of endothelial cells. Similar results were obtained in Matrigel invasion assay in Boyden chambers (Fig. 3c). Indeed, Exos-treated ECFCs and HMVECs showed an increase of invasion properties compared to untreated cells. Likewise, A375-Exo treatment enhanced ECFC and HMVEC proliferation and motility (migration and invasion) (Supplementary Fig. S3).

**Fig. 4:**
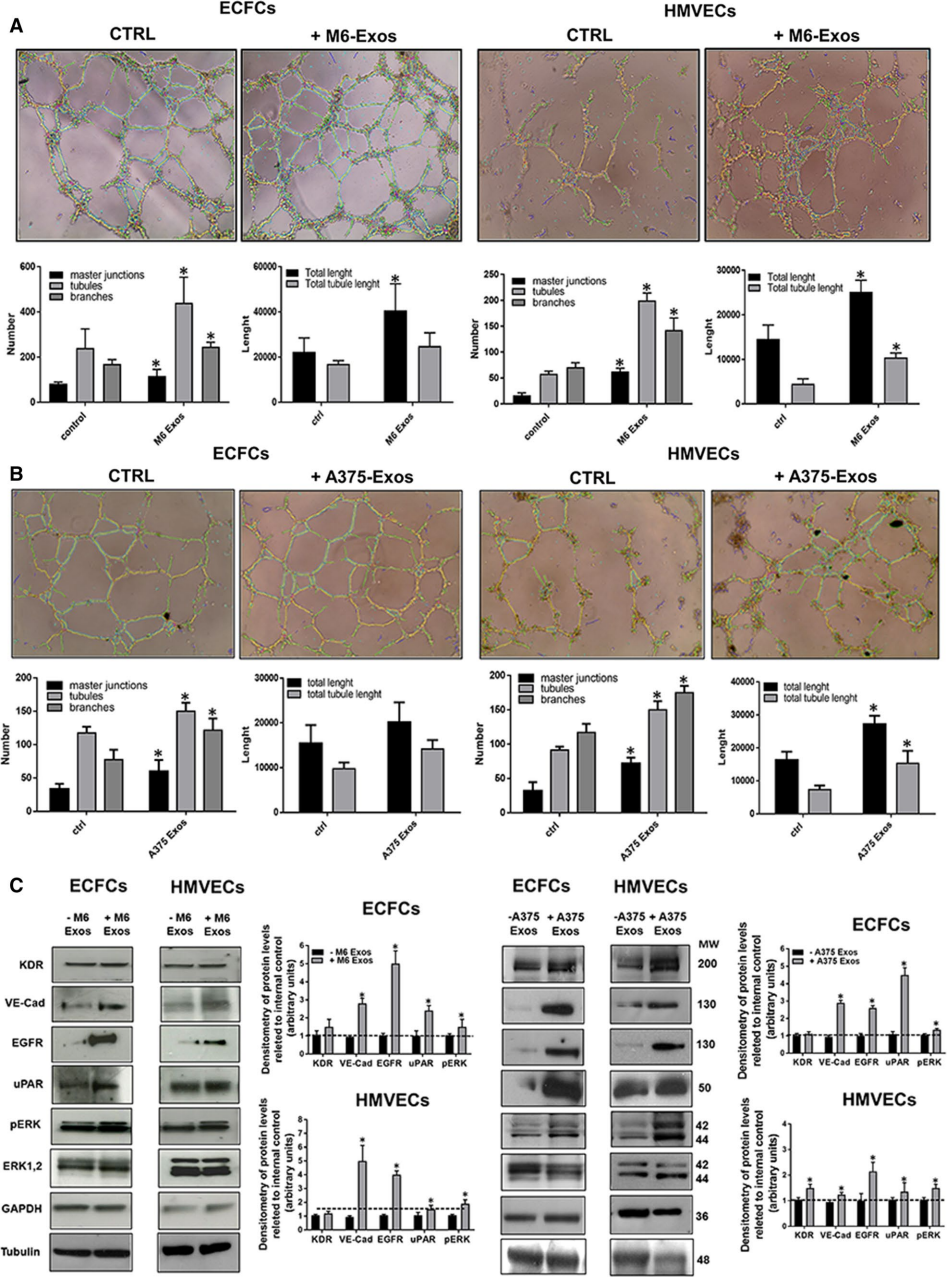
Effects of M6-Exos and A375-Exos on tube formation and angiogenesis-related signaling in ECFCs and HMVECs. Capillary morphogenesis assays in the presence or absence of M6-Exos (**a**) and A375-Exos (**b**). Representative microphotographs (× 10) of capillary-like structures are shown. Quantification of capillary network by Angiogenesis Analyzer Image J tool. Histograms represent the mean number of master junctions, branches, tubules, total length, and total tubule length, respectively. Data are representative of measures obtained from at least nine fields. * Shows statistical significance (*p* < 0.05) compared to untreated cells. **c** Western Blotting analyses of KDR, VE-Cad, EGFR, uPAR, pERK, ERK1,2. GAPDH and tubulin were used as a loading control. Densitometric quantification of the immunoblots normalized to the relative internal control and expressed respect to untreated (- M6 and A375 exos) ECFCs and HMVECs is reported on the right. *MW* molecular weight

**Fig. 5:**
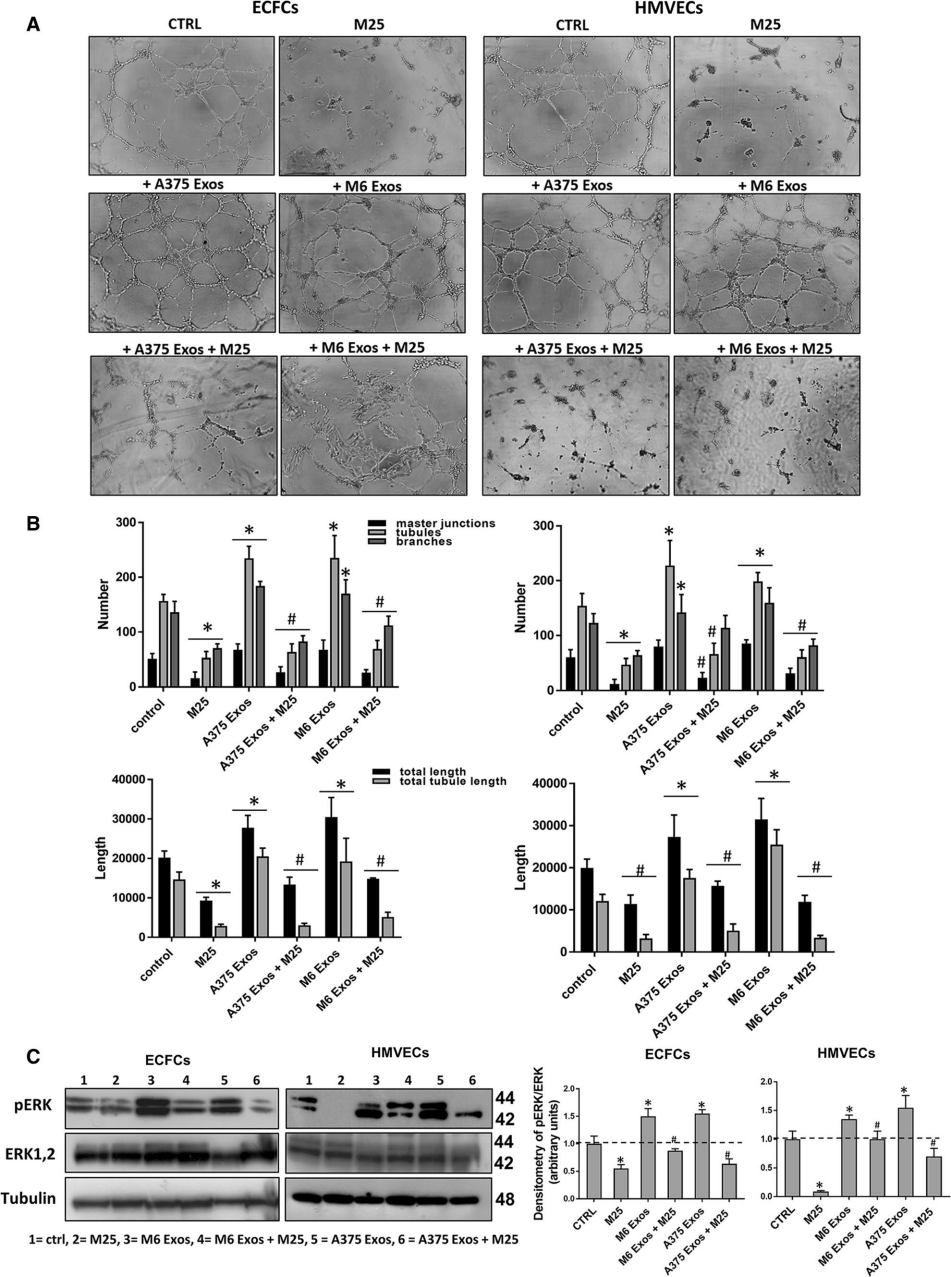
Activity of M25 peptide on the pro-angiogenic effects of melanoma- Exos. Capillary morphogenesis assays of M6-Exo and A375-Exo-treated ECFCs and HMVECs in the presence of absence of M25 peptide. **a** Representative microphotographs (× 10) of capillary-like structures are shown. **b** Quantification of capillary network by Angiogenesis Analyzer Image J tool. Histograms represent the mean number of master junctions, branches, tubules, total length, and total tubule length, respectively. Data are representative of measures obtained from at least nine fields. * Shows statistical significance (*p* < 0.05) compared to untreated cells. **c** Western Blotting analyses of p ERK and ERK1,2. Tubulin was used as a loading control. Densitometric quantification of the immunoblots normalized to the relative internal control and expressed respect to untreated (- M6 and A375 exos) ECFCs and HMVECs is reported on the right. *MW* molecular weight

**Fig. 6:**
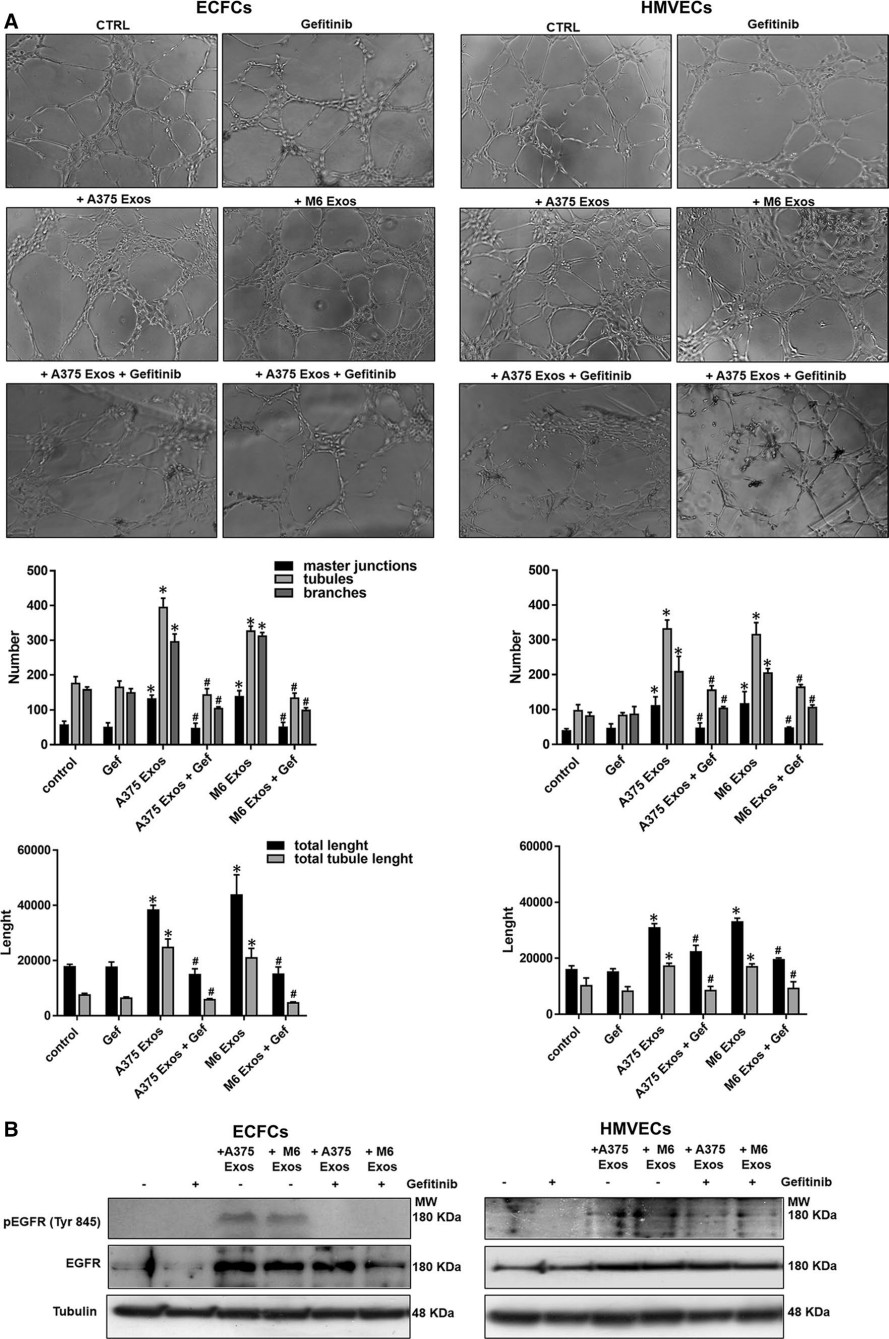
Anti-Exos pro-angiogenic activity of Gefitinib and inhibition of Exos-dependent EGFR phosphorylation. Capillary morphogenesis assays of M6-Exo and A375-Exo-treated ECFCs and HMVECs in the presence of absence of Gefitinib 10 µm. **a** Representative microphotographs (× 10) of capillary-like structures are shown. **b** Quantification of capillary network by Angiogenesis Analyzer Image J tool. Histograms represent the mean number of master junctions, branches, tubules, total length, and total tubule length, respectively. Data are representative of measures obtained from at least nine fields. * Shows statistical significance (*p* < 0.05) compared to untreated cells. **c** Western Blotting analyses of pEGFR and EGFR. Tubulin was used as a loading control. *MW* molecular weight

Lastly, to investigate the effect of A375- and M6-Exos on the angiogenic tubule formation, we performed capillary morphogenesis assays in Matrigel, in the presence or absence of A375- and M6-Exos. As shown in Fig. 4a and b, the number of master junctions, branches, tubules, total length e total tubule length were significantly increased in Exo-treated HMVECs and ECFCs and a better capillary network was observed compared to untreated cells. Therefore, taken together, our in vitro functional assays indicate that A375- and M6-Exos could activate a pro-angiogenic response in recipient endothelial cells. To investigate the mechanism through which A375- and M6-Exos could modulate endothelial cell function, we analyzed by Western Blotting the cell signaling transductions involved in angiogenesis. Figure 4c shows that Exos treatment induced a relevant increase of uPAR, EGFR and VE-Cadherin protein levels and a slight increment of KDR expression. These features were coupled with an increase of ERK1,2 (p42/44) phosphorylation in both ECFCs and HMVECs. Both morphological and signaling differences induced by A375- and M6-Exos were inhibited by the integrin antagonist peptide M25, that uncouples integrin-mediated uPAR–EGFR interaction, as previously shown [18] (Fig. 5, panels a, b and c). To prove the direct role of EGFR in Exo-mediated angiogenesis, we performed capillary morphogenesis in the presence or absence of Gefitinib, an EGFR inhibitor which interrupts the signaling through EGFR in target cells. As shown in Fig. 6 panel a, Gefitinib reduced the pro-angiogenic effects of A375- and M6-Exos in ECFCs and HMVECs. In parallel, in the presence of Gefitinib, we observed an inhibition of Exos-mediated EGFR phosphorylation. (Fig. 6 panel b). Taken together, these data indicate that the uPAR interactome transferred by melanoma exos in target endothelial cells could play a prominent role in melanoma-associated tumor angiogenesis by control of EGFR signaling.

### uPAR is required for the pro-angiogenic activity of A375- and M6-Exos

**Fig. 7:**
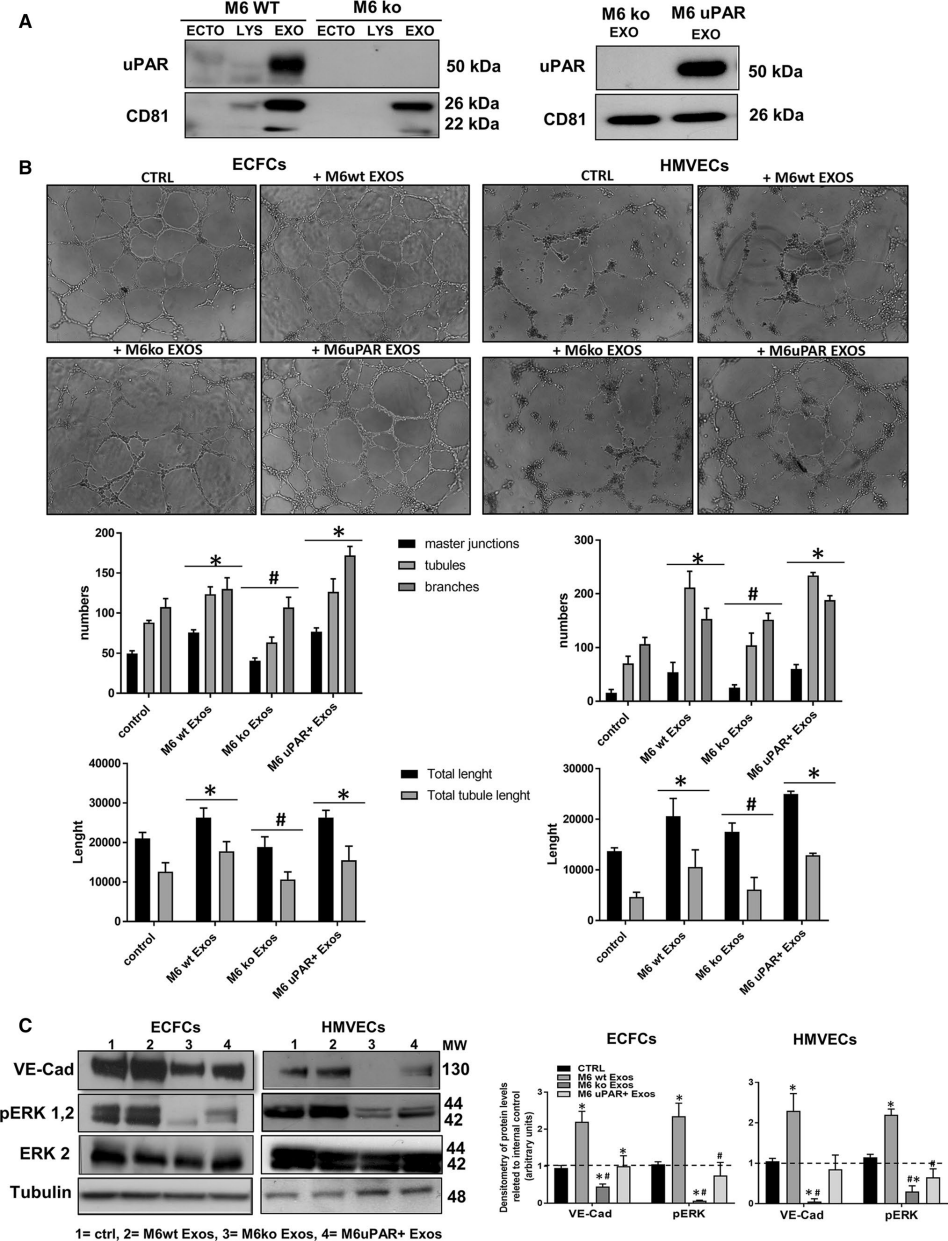
Effects of uPAR knockout on the pro-angiogenic activities of melanoma Exos. **a** Western Blotting analysis of uPAR in M6-ko and M6-wt Exos (on the left) and in M6-ko and M6 u PAR+Exos (on the right). CD81 was used as loading control for exosomal samples. **b** Capillary morphogenesis assays in the presence of M6 wild-type Exos (M6-wt Exos), M6 uPAR Ko Exos (M6 Ko Exos) and uPAR rescued-Exos (M6 uPAR+Exos). Upper part: representative microphotographs (× 10) of capillary-like structures are shown. Lower part: quantification of capillary network by Angiogenesis Analyzer Image J tool. Histograms represent the mean number of master junctions, branches, tubules, total length, and total tubule length, respectively. Data are representative of measures obtained from at least nine fields. * Shows statistical significance (*p* < 0.05) compared to untreated cells. # shows statistical significance (*p* < 0.05) compared to M6-wt Exos. **c** Western blotting analyses of VE-Cad, p ERK1,2, ERK1,2 and Tubulin in ECFCs and HMVECs in control conditions (ctrl) and treated with wild-type Exos (M6-wt Exos), M6 uPAR Ko Exos (M6 Ko Exos) and uPAR rescued-Exos (M6 uPAR+Exos). Densitometric quantification of the immunoblots normalized to the relative internal control and expressed respect to untreated (- M6 and A375 exos) ECFCs and HMVECs is reported on the right. *MW* molecular weight

To establish a direct correlation between uPAR expression in melanoma Exos and their pro-angiogenic effects, we performed siRNA-mediated uPAR silencing in M6. After validation of uPAR down-regulation by Western Blotting (Supplementary Fig. S4A), we purified Exos from siCONTROL and siPLAUR M6 CM, respectively. Supplementary Fig.S4B shows a consistent inhibition of capillary-like structures in siPLAUR-Exos-treated compared to siCONTROL-Exos-treated ECFCs. To further validate the evidence that uPAR is critical for the Exos-mediated angiogenesis, we have exploited the CRISPR–CAS9 technology to obtain a robust irreversible uPAR gene knockout in A375 and M6 (Fig. 7a, left panel), as recently published [29]. In parallel, to directly demonstrate the role of uPAR, we performed an experiment of expression rescue (Fig. 7a, right panel). Exos were collected from CM of uPAR ko and uPAR Rescue (uPAR+) A375 and M6 and capillary morphogenesis assays were performed. We observed a significant reduction of capillary network in the presence of uPAR ko-Exos both in HMVECs and in ECFCs that was restored in the presence of uPAR+ -Exos (Fig. 7b). The levels of VE-Cad and pERK1,2 (Fig. 6c) were remarkably reduced in ko-Exos-treated HMVECs and ECFCs compared to control and, on the other hand, increased in uPAR + -Exo-treated endothelial cells. Similar results were obtained in A375 (Supplementary Fig. S5). These results suggest that uPAR is critical for the pro-angiogenic effects of melanoma-derived Exos through a ERK1,2-mediated pathway.

### uPAR + melanoma Exos promote angiogenesis in vivo

**Fig. 8:**
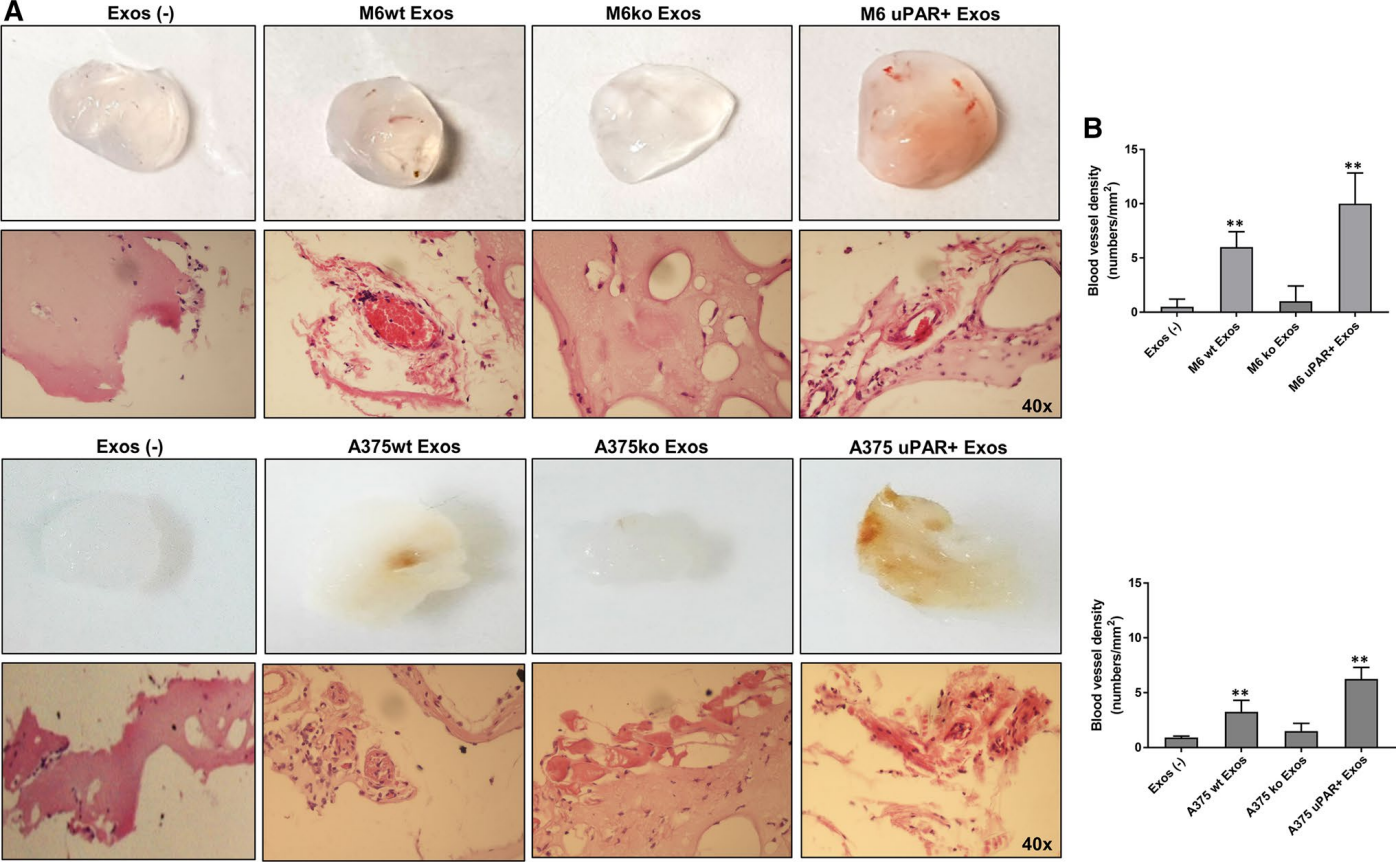
uPAR+melanoma exosomes promote angiogenesis in vivo. Matrigel plug assay: Matrigel containing wt, ko and uPAR+Exos, or not, were injected subcutaneously in the nude mice. Representative images of the Matrigel plugs were shown (Fig. 7a). In vivo neovascularization induced by M6- and A375-Exos was evaluated by Hematoxylin and Eosin staining and quantified by blood vessel density (Fig. 7b) using Image J software

To validate our observations in vivo, we further assessed the angiogenic potential of Exos by examining the recruitment of endothelial cells and vasculature formation within subcutaneously implanted Matrigel plugs containing Exos derived from wild type, uPAR ko and uPAR Rescue (uPAR+) M6 and A375. The plugs containing uPAR+ -Exos become more vascularized than implants with uPAR ko- and wild-type Exos (Fig. 8a). Histological examination by Hematoxylin and Eosin staining indicates that Matrigel plugs with uPAR+ -Exos showed more micro-vessels compare to plugs containing uPAR ko and wild-type Exos. Indeed, the vascular density of Matrigel plugs (Fig. 8b) with uPAR+ -Exos was significantly higher than those containing uPAR ko, that not contain blood vessels, and wild-type Exos. These data suggest that wild-type and uPAR+ -Exos play an evident role for endothelial cells recruitment and vascular organization in vivo. So, uPAR expression is important for the angiogenic potential of A375- and M6-Exos both in vitro and in vivo*.*

## Discussion

Accumulating evidence indicate that Exos are important in melanoma progression, supporting pro-tumoral processes including angiogenesis, immune regulation and modification of tumor microenvironment [11]. In addition, Exos may also vehiculate pro-angiogenic molecules that promote neo-angiogenesis and ECM remodeling [32]. The formation of a new vascular network and remodelling of the extracellular space are crucial for the detachment of melanoma cells from the primary site, which represents the first steps of the metastatic cascade.

In the present study, we have investigated the role of uPAR in Exos derived from melanoma cells and their pro-angiogenic effects in HMVECs and ECFCs. First, we have isolated Exos from A375 and M6, derived from a lung metastasis of A375, by ultracentrifugation and filtration. Then, we have characterized the purified Exos in terms of dimensions, integrity and the typical protein markers expression. A375- and M6-Exos appeared as round-, cup-shaped EVs, ranging from 50 to 150 nm, and expressing CD63, CD81, CD9 and Alix. These data are consistent with what has been reported for Exos from other melanoma cell lines [33]. In addition, we analyzed the expression of uPAR, an important prognostic and predictive marker of malignancy in melanoma cells [14, 16]. Here, we demonstrated that uPAR is expressed in Exos but not in ectosomes. A possible explanation of this differential expression may be found in uPAR localization in specialized membrane microdomains, called lipid rafts. We have previously shown that the angiogenic properties of ECFCs depend on the integrity of caveolae and on the presence of full-length uPAR in such structures [24]. We have also demonstrated that uPAR binds preferentially to ganglioside GM1-enriched membranes, promoting invasion and capillary morphogenesis in ECFCs [34]. As reported by Thuma F. et al. [35], uPAR associates with palmitoylated claudin7, a major component of tight junctions, located in tetraspanin-enriched microdomains, similar to glycolipid-enriched microdomains (GEM). These specialized membrane regions, prone for internalization [36, 37], are recruited into early endosomes and these complexes are maintained and recovered in Exos [38–40]. In addition, Endo-Munoz L et al. [41] have observed that, in metastatic osteosarcoma, uPA was secreted in an active Exos-bound form, influencing metastatic behavior via locally secreted uPA and at distant sites via uPA-containing Exos. Therefore, it is likely that uPAR may reside in Exos rather than in ectosomes. After their characterization, we studied the ability of melanoma Exos to stimulate an angiogenic program in endothelial cells. We have shown that A375- and M6-Exos are internalized and are able to promote the angiogenic properties of endothelial cells stimulating in vitro proliferation, migration, invasion and capillary-like structure formation both in HMVECs and ECFCs. Considering the effect of A375 and M6-Exos in vivo, we observed that Exos from both parental cell lines promote endothelial cells recruitment and vascular organization within Matrigel plugs*.*

The uPA/uPAR system is essential for the endothelial function and for a correct angiogenic program. Indeed, we have previously reported that full-length uPAR is required for the angiogenic response of ECFCs [24]. At the same time, we have also shown that the MMP12-dependent uPAR cleavage is responsible for an angiogenesis impairment in HMVECs [42]. Here, to study the uPAR function in Exos, we exploited the CRISPR–Cas 9 technology to obtain a complete uPAR knockout, as recently published [29]. In this study, we reported that the uPAR silencing and, even more, the CRISPR-mediated knockout abrogate the pro-angiogenic potential of melanoma Exos both in vitro and in vivo. On the other hand, the pro-angiogenic ability of melanoma Exos is recovered by uPAR rescue, demonstrating a direct correlation between exosomal expression of uPAR and the pro-angiogenic properties of Exos.

During angiogenesis, adhesion molecules are important to provide connections between endothelial cells and to maintain the integrity of vascular tubes [43, 44]. The Vascular Endothelial Cadherin (VE-Cadherin) is a key adhesion molecule actively involved in the formation and stabilization of intercellular adherens junctions in endothelial cells and in modulating signaling cascades within endothelial cells during angiogenesis, vessel morphogenesis and vascular development [44]. Accordingly, also VEGF/VEGFR2 interaction activates VE-Cadherin expression and the signaling pathway ERK/MAPK [45]. Here we have shown that the A375- and M6-Exos treatment induced an increase of VE-Cadherin, uPAR and EGFR protein levels both in mature and progenitor endothelial cells, in parallel with an increment of ERK1,2 phosphorylation, a signaling pathway that is inhibited by the peptide M25 which uncouples integrins-dependent uPAR interactions with receptor tyrosine kinases (RTKs), including EGFR [17, 18]. These data are in agreement with the paper of LaRusch GA et al. in which a β1-integrin peptide that binds uPAR blocks FXII-induced angiogenesis inhibiting the ERK1,2 and Akt phosphorylation in human umbilical vein endothelial cells (HUVEC) [46].

In the presence of Exos from uPAR knockout parental cell lines, we observed a reduction of VE-Cadherin, and uPAR and EGFR expression, in parallel with a decrease of ERK1,2 phosphorylation. Again, VE-Cadherin, uPAR and EGFR levels and ERK1,2 signaling were restored in endothelial cells after uPAR rescue. Our data on Exos-dependent endothelial cell expression of EGFR, its phosphorylation and Gefitinib-dependent inhibition of Exos-induced capillary morphogenesis (Fig. 6) are supported by several evidences that stimulation or inhibition of EGFR has significant consequences on tumor angiogenesis, a feature that involves both a direct effect and an interplay with VEGF [47–51]. In light of our results, it could be speculated that the reported abundance of EGFR (Erb-B1) on endothelial cells of tumor vessels [49, 50] could depend on an enrichment mediated by the release of Exos by the malignant cells.

Moreover, the crosstalk between uPAR and VE-Cadherin has been previously shown. Brunner et al. observed that in initial phases of angiogenesis, uPAR undergoes down-regulation by density-enhanced phosphatase-1 (DEP-1) in confluent endothelial cells [52]. The up-regulation of DEP-1, with increasing cell density, inhibits the ERK1,2 pathway and uPAR expression in confluent endothelial cells, through VE-Cadherin/B-catenin interaction [53].

Collectively, our findings demonstrate that uPAR is critical for the Exos-mediated angiogenic program in human malignant melanoma and that the evaluation of exosomal uPAR expression and the complete uPAR knockout by gene editing technique may be a potential approach for monitoring and treatment of human melanoma. As a future perspective, this study provides new insights for a possible use of uPAR as a helpful biomarker in exosomal preparations obtained from the liquid biopsy in metastatic melanoma patients.

### Electronic supplementary material

Below is the link to the electronic supplementary material.

Supplementary file1 Figure S1. Characterization of A375-derived Exos (TIF 7921 KB)

Supplementary file2 Figure S2. Internalization of A375-Exos into ECFCs and HMVECs (TIF 9661 KB)

Supplementary file3 Figure S3. Effects of A375-Exos on proliferation, migration and invasion of ECFCs and HMVECs. (TIF 9879 KB)

Supplementary file4 Figure S4. Effects of uPAR siRNA-mediated silencing on the pro-angiogenic activities of M6-Exos. (TIF 9001 KB)

Supplementary file5 Figure S5. Effects of wt, uPAR ko and uPAR+ A375-Exos on angiogenic properties of ECFCs and HMVECs and western blotting analyses of VE-Cad and pERK1,2. (TIF 6306 KB)

Supplementary file6 (DOCX 18 KB)

## Data Availability

All the data generated or analyzed during this study are included in this published article and its supplementary files. The datasets and materials in the current study available from the corresponding author on reasonable request.

## Abbreviations

EVs: Extracellular vesicles
MVs: Microvesicles
Exos: Exosomes
MVB: Multivesicular bodies
uPA: Urokinase plasminogen activator
uPAR: Urokinase plasminogen activator receptor
PAI-1: Plasminogen activator inhibitor 1
EGFR: Epidermal growth factor receptor
suPAR: Soluble plasminogen activator receptor
ECFCs: Endothelial colony-forming cells
HMVEC: Human microvascular endothelial cells
CRISPR: Clustered regularly interspaced short palindromic repeats
TRPS: Tunable resistive pulse sensing
LAMP-1: Lysosomal-associated membrane protein 1
CM: Culture media
siRNA: Small interfering RNA
VE-Cad: Vascular endothelial cadherin
ECM: Extracellular matrix
MMP-12: Matrix metallo-proteinase-12
