# Supplementary figures and images for: uPAR-expressing melanoma exosomes promote angiogenesis by VE-Cadherin, EGFR and uPAR overexpression and rise of ERK1,2 signaling in endothelial cells
Source: Cell Mol Life Sci. 2020 Nov 25;78(6):3057–72. doi: 10.1007/s00018-020-03707-4 (PMC8004497; doi:10.1007/s00018-020-03707-4)

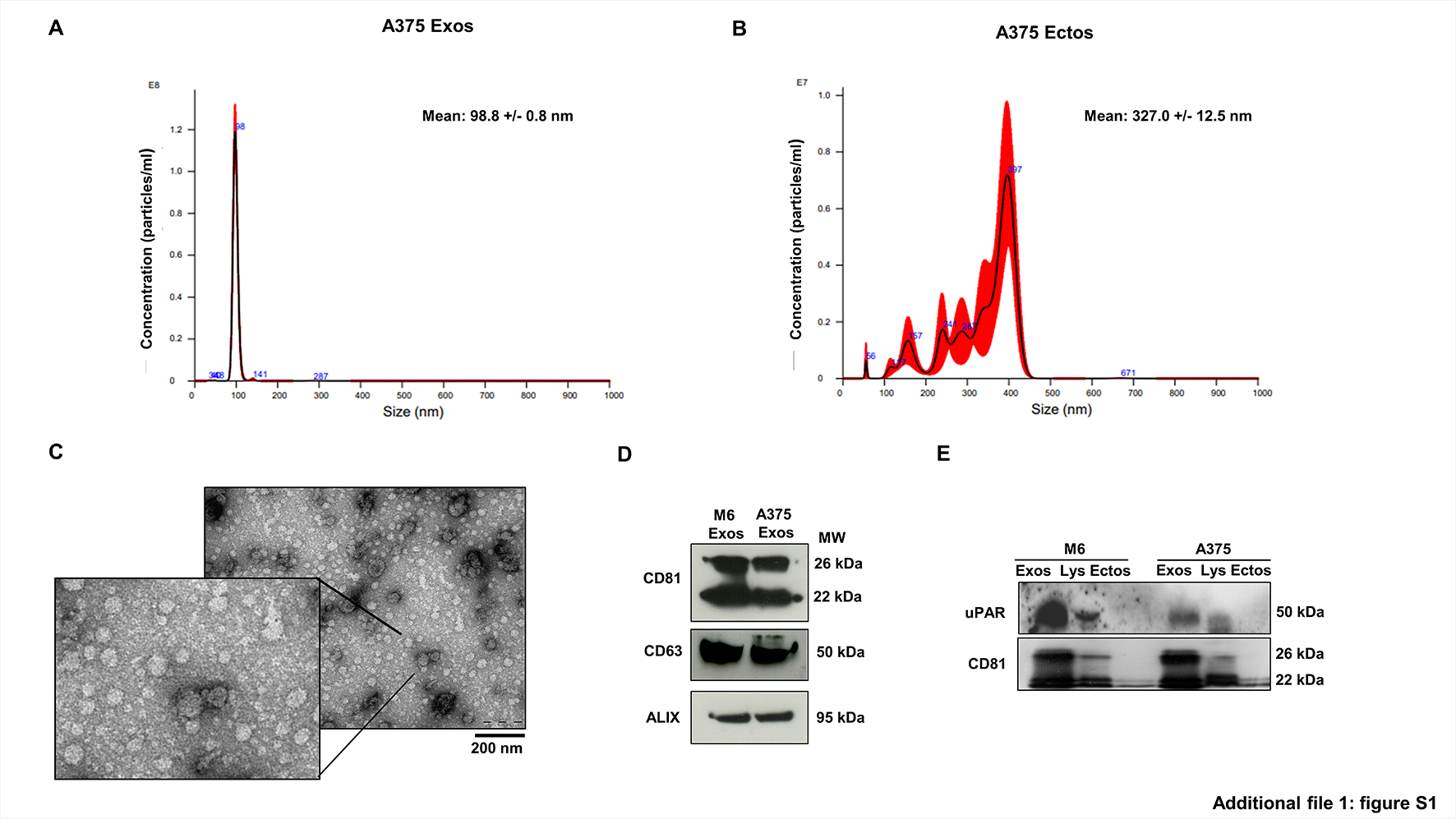

Supplement: Supplementary file 1 — Supplementary file1 Figure S1. Characterization of A375-derived Exos (TIF 7921 KB) [file 18_2020_3707_MOESM1_ESM.tif]

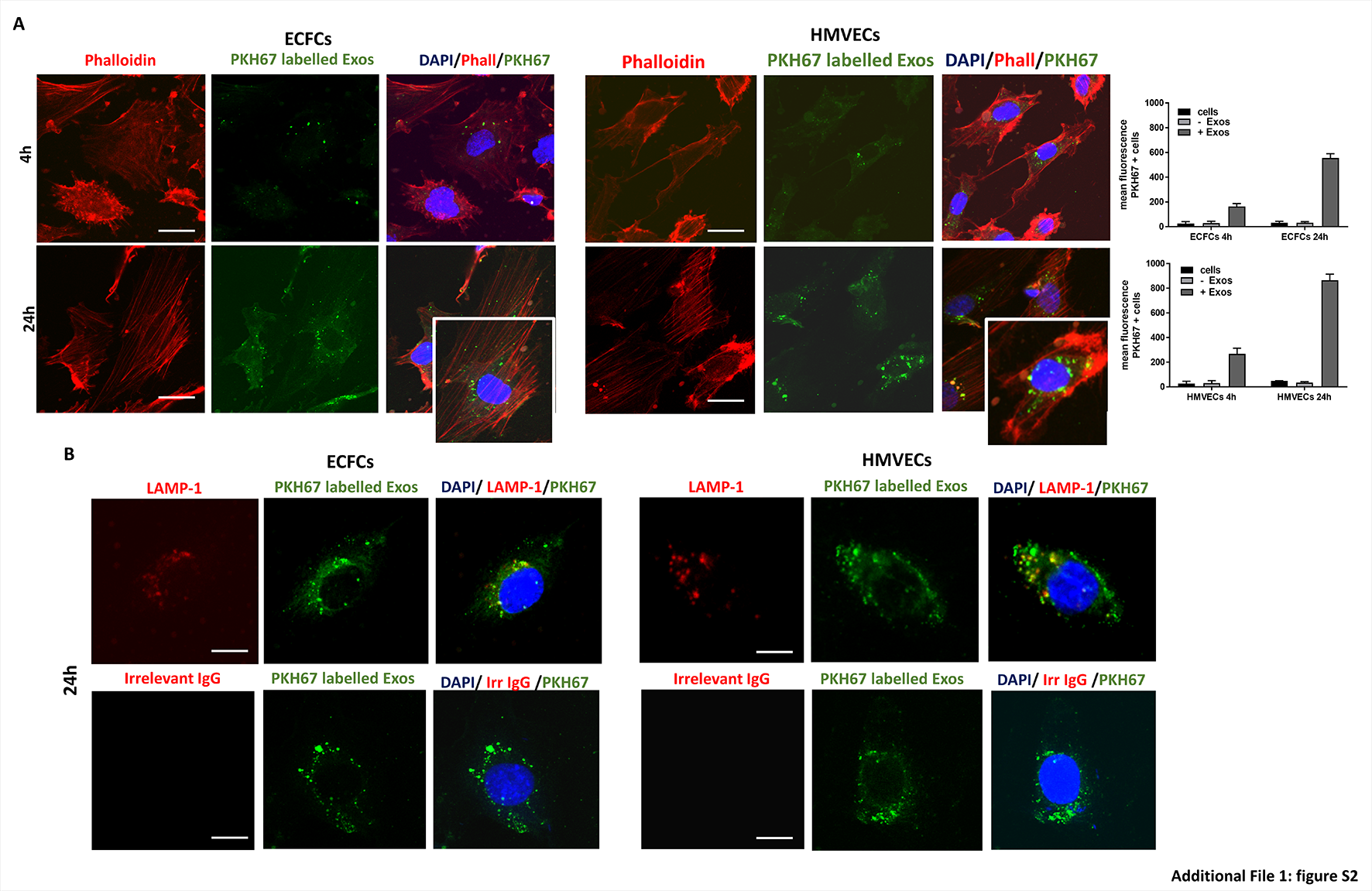

Supplement: Supplementary file 2 — Supplementary file2 Figure S2. Internalization of A375-Exos into ECFCs and HMVECs (TIF 9661 KB) [file 18_2020_3707_MOESM2_ESM.tif]

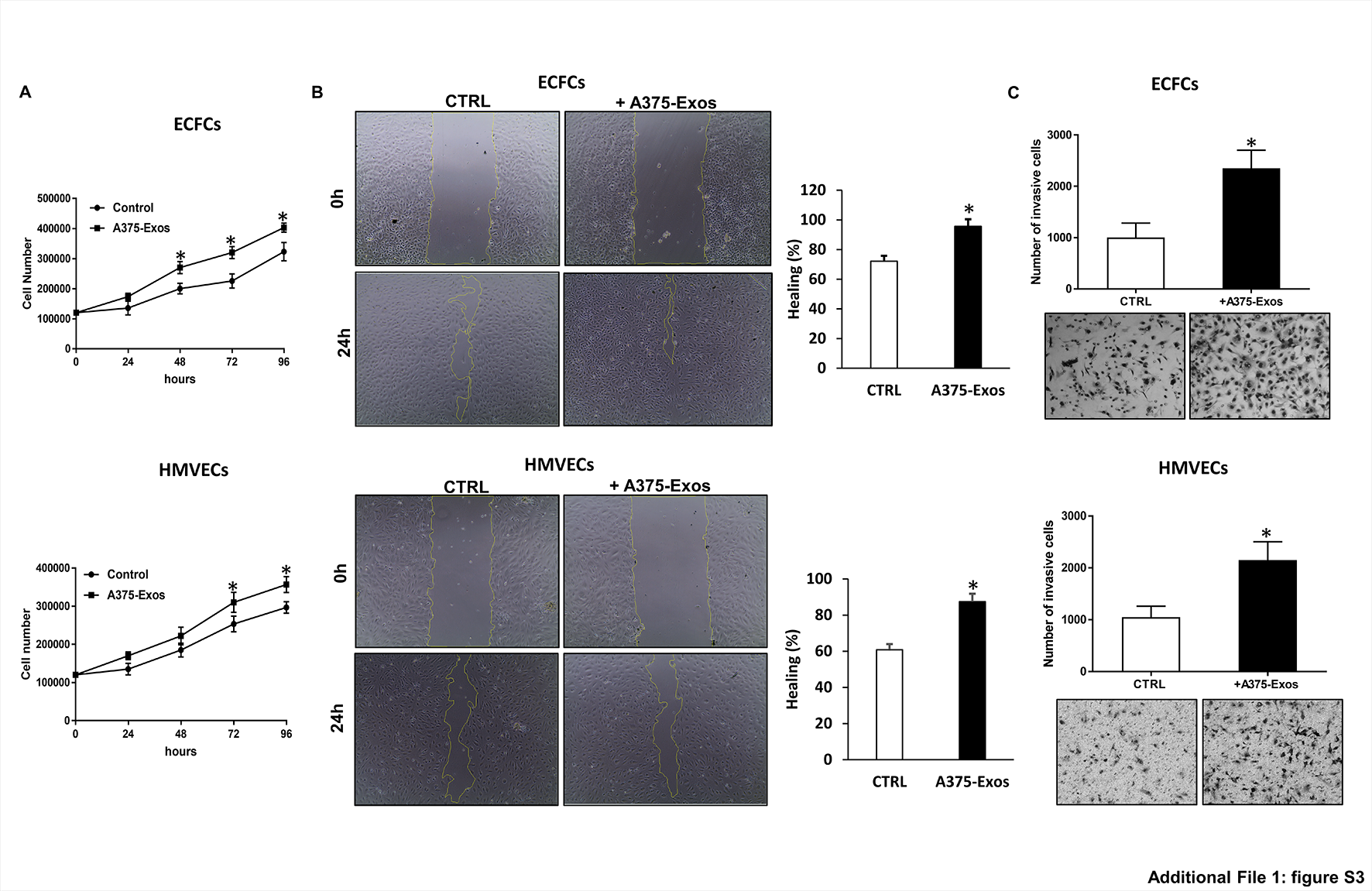

Supplement: Supplementary file 3 — Supplementary file3 Figure S3. Effects of A375-Exos on proliferation, migration and invasion of ECFCs and HMVECs. (TIF 9879 KB) [file 18_2020_3707_MOESM3_ESM.tif]

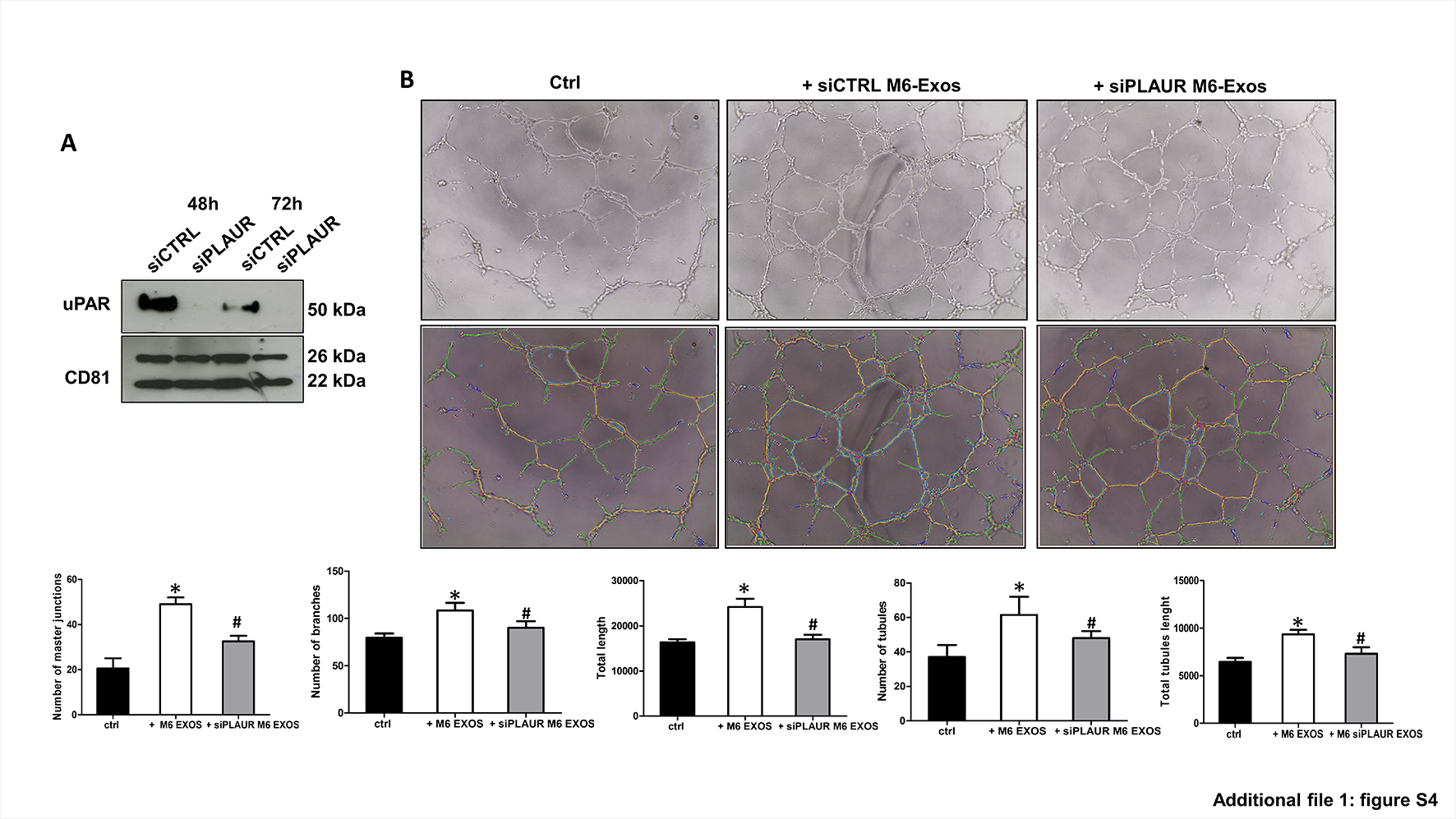

Supplement: Supplementary file 4 — Supplementary file4 Figure S4. Effects of uPAR siRNA-mediated silencing on the pro-angiogenic activities of M6-Exos. (TIF 9001 KB) [file 18_2020_3707_MOESM4_ESM.tif]

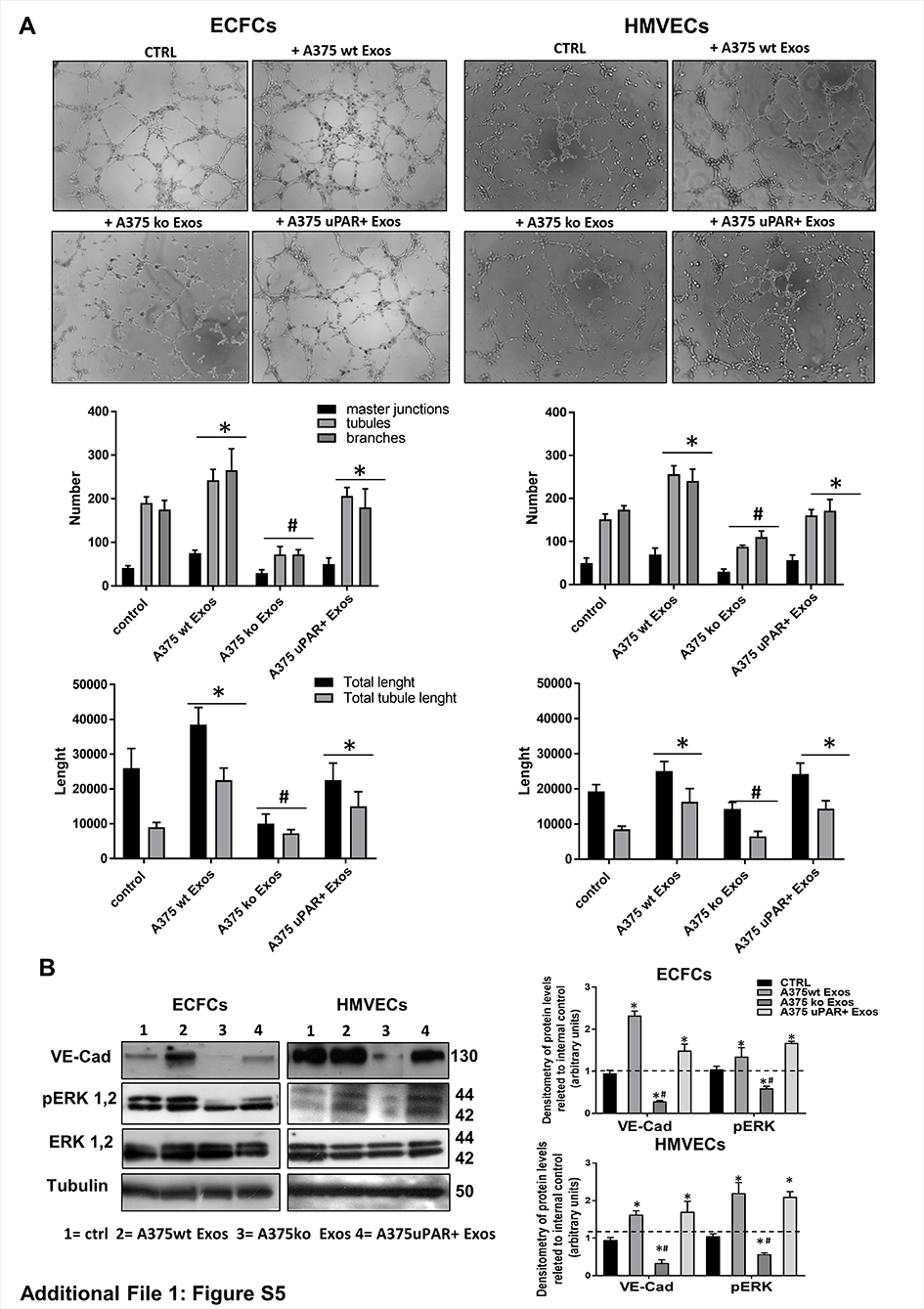

Supplement: Supplementary file 5 — Supplementary file5 Figure S5. Effects of wt, uPAR ko and uPAR+ A375-Exos on angiogenic properties of ECFCs and HMVECs and western blotting analyses of VE-Cad and pERK1,2. (TIF 6306 KB) [file 18_2020_3707_MOESM5_ESM.tif]
