# Supplementary material for: uPAR-expressing melanoma exosomes promote angiogenesis by VE-Cadherin, EGFR and uPAR overexpression and rise of ERK1,2 signaling in endothelial cells
Source: Cell Mol Life Sci. 2020 Nov 25;78(6):3057–72. doi: 10.1007/s00018-020-03707-4 (PMC8004497; doi:10.1007/s00018-020-03707-4)
Supplement: Supplementary file 6 — Supplementary file6 (DOCX 18 KB) [file 18_2020_3707_MOESM6_ESM.docx]

**SUPPLEMENTARY METHODS**

**Exosomes isolation**

Exos isolation protocol was modified from previously published methods (25,26). 80 ml of cell culture supernatant collected from A375 and M6 was centrifuged at 300 g for 5 min (Heraeus 11R, Thermo Scientific) to discard the cell pellet. The supernatant was transferred to a fresh 50 ml tube and centrifuged at 2600 g for 15 min to remove cell debris and apoptotic bodies. The supernatant was then filtered using a 0.20 μm and 0.10 μm syringe filter (Millex, Millipore) to remove remaining cells, debris and EVs larger than Exos. The resultant supernatant was isolated for ectosomes and Exos by ultracentrifugation. Briefly, supernatant was ultracentrifuged at 12.000 g for 20 min at 4 °C (SW32 Ti Rotor, Swinging Bucket in Beckman Coulter 90K Ultracentrifuge) for ectosome and then at 100.000 g per 1h a 4 °C for Exos collection. The ultracentrifuged supernatant was carefully decanted and the pellet was suspended in an appropriate volume of filtered PBS.

**PKH67 labeling of Exos and exosomal uptake into recipient cells**

Melanoma-derived Exos were collected from 80 ml of culture medium as described above. Exos were labeled using PKH67 Fluorescent Cell Linker kits (Sigma-Aldrich, St. Louis, MO) according to the manufacturer's instructions. After washing, exosomal pellets were resuspended in 250 μl of Diluent C (exosomal solution). One microliter of PKH67 dye was diluted in 250 μl of Diluent C to prepare the PKH67 solution, and 250 μl of the exosomal solution and 250 μl of the PKH67 solution were mixed. Then, 1ml of 1% bovine serum albumin was added to bind any excess PKH67 dye. PKH67-labeled Exos were ultracentrifuged at 100,000 × g at 4°C for 1h. Exos pellets were washed three times in PBS by ultracentrifugation at 100,000 × g at 4°C for 1h and resuspended in 100 μl PBS. To examine the uptake of Exos into recipient endothelial cells, HMVECs and ECFCs were plated in the coverslips into 6-well plates at a density of 1×10^4^ cells per well. After 24 h, the cells were washed three times in PBS, and EBM-2 containing either PKH67-labeled exosomal solution or control solution was added to each well. Cells were cultured for 4 and 24h at 37°C in a humidified atmosphere with 5% CO2. The slides were washed three times in PBS and fixed with 3.7% formaldehyde solution at room temperature for 20 min. Slides were then washed three times in PBS. In parallel, to measure the uptake of exosomes cells after 4h and 24h cells were washed three times with PBS, trypsinized and resuspended in 500 μl of PBS. Exos uptake was measured on a FACSCAN LSRII (Becton-Dickinson, excitation = 490 nm, emission = 502 nm) as previously described [27].

**Immunofluorescence confocal microscopy**

Immunofluorescence was performed as previously described (24,27). Fixed HMVECs and ECFCs were stained with TRITC-labelled phalloidin (P1951, Sigma) to visualize cell morphology and with anti-LAMP-1 (Lysosome-associated membrane protein-1) (Cell Signaling Technology, USA), a lysosomal marker to analyze Exos localization. Cy3–conjugated goat anti-rabbit IgG (Sigma–Aldrich Chemicals) was used as secondary antibody. Nuclei were stained with the 4',6-diamidino-2-phenylindole (DAPI) (10µg/ ml) (Invitrogen) for 15 min at room temperature. A single composite image was obtained by superimposition of twenty optical sections for each sample observed by confocal laser scanning microscope (Nikon).

**Invasion assays in Boyden chambers**

Spontaneous invasion experiments were performed in Boyden chambers with wells separated by 8µm-pore size polycarbonate filters coated with Matrigel (50µg/filter). ECFC and HMVEC cultures were detached, counted and then resuspended in 200 µL of EBM supplemented with 2% FBS and placed in the upper well of Boyden chambers in presence or absence of A375-Exos and M6-Exos (20 µg/ml). Fresh EBM plus 2% FBS was placed in the lower well. Invasion was performed for 6 hours at 37°C in 5% CO_2_, then filters were recovered and fixed in methanol. Non-invading cells on the upper surface of the filter were removed with a cotton swab while invasive cells adherent on the lower filter surface were stained and counted using a light microscope. Results were reported as the number of ECFCs and HMVECs migrated after incubation with A375-Exos and M6-Exos compared to those migrated after incubation with EBM.

**Western Blot analyses**

Ectosome, Exos and cell aliquots of A375 and M6, as well as cell aliquots of control and Exos-treated ECFC and HMVEC cultures were collected at the end of the respective treatments and lysed in RIPA buffer (25mM Tris–HCl pH 7.6, 150mM NaCl, 1% NP-40, 1% sodium deoxycholate, 0.1% sodium dodecyl sulfate, SDS) with 1% protease inhibitor Cocktail (Sigma-Aldrich) and disrupted by sonication (Microson XL-2000, Misonix). Lysates were clarified by centrifugation and supernatant collected and stored at -20°C. Protein content was measured trough Bradford assay. Protein aliquots (25-40µg) and ectosome/Exos samples, were separated by 12% SDS-polyacrylamide gel electrophoresis (SDS-PAGE, NuPAGE, Novex; Invitrogen), transferred to nitrocellulose membranes (Millipore), blocked in 5% skim milk and incubated overnight with the specific primary antibodies: mouse anti-Alix (Santa Cruz), mouse anti-CD63 (Santa Cruz), mouse anti-CD81 (Santa Cruz), mouse anti-CD9 (Santa Cruz) mouse anti-uPAR (R3) (Thermo Fisher) which recognizes the full-length uPAR, rabbit anti-phospho-ERK (p42/p44, Cell Signaling), rabbit anti-phospho-EGFR (Tyr 845) (Cell Signaling), rabbit anti-EGFR (Santa Cruz), rabbit anti-ERK (p42/p44, Santa Cruz), rabbit anti-VE-Cadherin (Santa Cruz), rabbit anti-KDR (Cell Signaling ), rabbit anti-GAPDH (Cell Signaling), anti-mouse Tubulin (Sigma Aldrich) followed by the suitable HRP-coniugated secondary antibodies (Sigma–Aldrich). All the resulting immunocomplexes were visualized with an enhanced chemiluminescence ECL detection system (GE Healthcare) and quantified by ImageJ software (NIH).

**Cell treatment with M25 integrin antagonist peptide and Gefitinib**

Inhibition of uPAR-integrin interaction was obtained with the M25 peptide, previously identified in a phage display library, able to uncouple uPAR from integrin α-chain. The peptide was produced in collaboration with the Peptide Facility at Biotechnology Center, University of Padova (CRIBI). In the β-propeller model of α-chain folding, the sequence of this peptide (STYHHLSLGYMYTLN) spans an exposed loop on the ligand-binding surface of α-chain, thus impairing integrin α chain-uPAR interaction. In cell culture M25 water solution was used at 50 μM at 37 °C. EGFR inhibition was obtained with Gefitinib used at 10 μM.

**siRNA for uPAR gene knock-down**

Targeting and not-targeting siRNAs were obtained from Dharmacon (Carlo Erba Reagents, Milan, Italy). Specific silencing of uPAR gene was performed by transfection of M6 with small-interfering-RNA (siGENOMESMARTpool), according to the manufactures' instructions. Not-targeting and GAPDH siRNA pool constructs were used as negative (siCONTROL) and positive control (siGAPDH) respectively. To favor cell internalization siRNAs were incorporated into cationic liposomes, utilizing DharmaFECT transfection reagent. Cells were incubated with transfection mix (24–48 h for mRNA analysis and 48-72 h for protein and phenotypic analysis, respectively).
